# Facilitators, barriers, and guidance to successful implementation of multidisciplinary transitional care interventions: A qualitative systematic review using the consolidated framework for implementation research

**DOI:** 10.1016/j.ijnsa.2024.100269

**Published:** 2024-11-29

**Authors:** Romain Collet, Juul van Grootel, Marike van der Leeden, Marike van der Schaaf, Johanna van Dongen, Suzanne Wiertsema, Edwin Geleijn, Mel Major, Raymond Ostelo

**Affiliations:** a Amsterdam UMC location Vrije Universiteit Amsterdam, Department of Rehabilitation Medicine, de Boelelaan 1117, Amsterdam, The Netherlands; b Amsterdam Movement Sciences, Musculoskeletal Health, Amsterdam, The Netherlands; c Department of Health Sciences, Faculty of Science, Vrije University Amsterdam, The Netherlands; d Department of Epidemiology and Biostatistics, Amsterdam UMC, Vrije Universiteit Amsterdam, The Netherlands; e Amsterdam UMC location University of Amsterdam, Department of Rehabilitation Medicine, Meibergdreef 9, Amsterdam, The Netherlands; f Amsterdam Movement Sciences, Ageing and Vitality, Amsterdam, The Netherlands; g Faculty of Health, Center of Expertise Urban Vitality, Amsterdam University of Applied Sciences, The Netherlands; h Faculty of Health, Department of Physical Therapy, Amsterdam University of Applied Sciences, The Netherlands

**Keywords:** Continuity of patient care, Transitional care, Multimorbidity, Systematic review, Implementation science, Health personnel

## Abstract

**Background:**

Multidisciplinary transitional care interventions aim to improve the coordination and continuity of healthcare during hospitalization and after discharge for patients with complex care needs related to physical, nutritional, or psychosocial status. Implementing such interventions is complex as they involve many stakeholders across multiple settings. Numerous studies have evaluated patients’, family members’, and healthcare professionals’ experiences with multidisciplinary transitional care interventions, which can provide insight into facilitators and barriers to their implementation.

**Objective:**

To provide an overview of facilitators and barriers to implementing multidisciplinary transitional care interventions, which could be considered before developing implementation strategies.

**Design:**

A qualitative systematic review using the Consolidated Framework for Implementation Research.

**Setting(s):**

Hospitals and primary care

**Participants:**

Adult patients admitted to a hospital, regardless of their diagnosis, as well as their family members and hospital and primary care healthcare professionals

**Methods:**

Embase, CINAHL, and Medline were searched for qualitative studies evaluating multidisciplinary transitional care interventions through patients', family members', and healthcare professionals' experiences and views from inception until June 2024. The methodological rigor was assessed with the Critical Appraisal Skills Program. We identified facilitators and barriers to the successful implementation of multidisciplinary transitional care interventions with the Consolidated Framework for Implementation Research. Facilitators and barriers were categorized into pre- or post-discharge or general factors.

**Results:**

Twelve studies were included and appraised. We identified 79 factors, mostly linked to three domains of the Consolidated Framework for Implementation Research: Innovation, Inner setting, and Individuals involved. Facilitators included "comprehensive follow-up care needs assessment"(pre-discharge), "immediate, tailored follow-up care"(post-discharge), and "improved communication between stakeholders"(general). Barriers included "shortage of hospital beds" and "lack of time"(pre-discharge), "lack of available primary care professionals"(post-discharge), "inconsistencies of stakeholders' schedules" and "intervention costs"(general).

**Conclusions:**

The factors identified could serve as a non-exhaustive inventory list to inspire readers who wish to implement a multidisciplinary transitional care intervention in their settings. Digital tools and alternative financing models might overcome cost and reimbursement issues, the increasing complexity of patient care, and shortcomings, such as the lack of available hospital beds or professionals. Further research should identify effective implementation strategies, considering the pre-, post-discharge, and general factors identified.

**Registration:**

The protocol was registered in PROSPERO (CRD42023421423).

**Tweetable abstract:**

Effective communication aids in implementing transitional care interventions, but patient care complexity and healthcare system pressures present challenges.

**Contribution of the Paper**

What is already known

• The aging population and the increasing number of patients with complex care needs may result in unsustainable healthcare expenses in the coming years.
• Patients with complex care needs have difficulty navigating community care services on their own after hospital discharge, and effective multidisciplinary transitional care interventions that simultaneously address their medical, nursing, and allied healthcare needs are needed.
• Implementing multidisciplinary transitional care interventions is challenging because they involve many stakeholders across multiple settings, although many hospitals and primary care settings may wish to implement them, given their promising economic benefits and potential improvements in patient care.

What this paper adds

• Multidisciplinary transitional care interventions enable early identification of patients' follow-up care needs pre-discharge and provide tailored follow-up care in the community post-discharge through improved communication between stakeholders.
• The main factors hindering the implementation of these interventions were intervention costs not covered by health insurance, a shortage of hospital beds, a lack of available professionals in the community, and inconsistencies between stakeholders' schedules.
• While individual qualitative studies offer insights into a limited number of factors related to the successful implementation of multidisciplinary transitional care interventions, this review provides a comprehensive overview of potential systemic, organizational, and individual factors.

Alt-text: Unlabelled box

## 1 Introduction

The aging population and the increasing number of patients with multimorbidity have increased the complexity of care delivery during hospital-to-home transitions. As of 2023, the global prevalence of multimorbidity has been estimated to be 37.2 %, with evidence indicating a strong association between aging and multimorbidity (Chowdhury et al., 2023; Tazzeo et al., 2023). Delivering care to older adults or individuals with multimorbidity is particularly challenging, as they often have more complex care needs related to their physical, nutritional, cognitive, and/or psychosocial status due to interactions between many factors in their socio-ecological environment and inherited biology (e.g., lack of social support, genetic predispositions, psychological or emotional dysregulation, inappropriate utilization of healthcare services) (Sturmberg et al., 2021). In fact, both individuals aged 65 and older and those with multimorbidity have been shown to have a higher risk of hospital readmission (Aubert et al., 2019; Rodrigues et al., 2022).

*Multidisciplinary transitional care interventions* aim to improve the coordination and continuity of healthcare for patients transitioning from hospital to home by addressing patients’ complex care needs, that is, a combination of medical, nursing, and allied healthcare needs — such as physiotherapy, occupational therapy, and speech and language therapy (Naylor and Keating, 2008). These interventions generally involve hospital and primary care professionals collaborating to provide tailored care (Tyler et al., 2023). Several studies have shown their effectiveness in reducing hospital readmissions and emergency department visits and improving health-related outcomes (Coskun and Duygulu, 2022; Liu et al., 2023; Markle-Reid et al., 2023). Given the promising benefits of multidisciplinary transitional care interventions in enhancing patient care delivery and health-related outcomes, many hospitals may wish to implement them. However, implementing such interventions is challenging, because they involve many stakeholders across multiple settings.

Several qualitative studies have explored user experiences with multidisciplinary transitional care interventions (Allen et al., 2022; Kokorelias et al., 2023; Maximos et al., 2024); however, no comprehensive synthesis of the factors that facilitate or hinder the implementation of these interventions exists. While these individual qualitative studies offer insight into a limited number of factors contributing to the (non-)success of multidisciplinary transitional care interventions, a systematic synthesis of all available qualitative data can provide a thorough understanding of all potential systemic, organizational, and individual factors. The Consolidated Framework for Implementation Research is widely used to identify facilitators and barriers to successfully implementing healthcare interventions (Damschroder et al., 2022). For example, a recent qualitative study using this framework identified multiple key barriers and facilitators to implementing a multidisciplinary transitional care intervention through interviews with older adults, family caregivers, and stakeholders from healthcare support services (McAiney et al., 2022). Factors such as tailoring interventions to patient needs, patient engagement, and provider training were highlighted using the Consolidated Framework for Implementation Research.

Similarly, a comprehensive overview of all potential facilitators and barriers to implementing multidisciplinary transitional care interventions can be obtained by synthesizing and mapping data from all existing qualitative studies on the experiences of patients, family members, and healthcare professionals with these interventions. Therefore, this systematic review aimed to provide such an overview, offering valuable insights to guide researchers and clinicians in developing effective implementation strategies to enhance the success of multidisciplinary transitional care interventions in their settings.

## 2 Methods

The protocol of this review was registered in PROSPERO (CRD42023421423). We conducted a secondary analysis of studies previously included in a larger qualitative meta-synthesis, which comprised 53 qualitative articles investigating patients', family members’, and healthcare professionals’ experiences and needs regarding hospital-to-home transitions (van Grootel et al., 2024). The larger meta-synthesis included qualitative studies on experiences around the hospital-to-home transition in general, which might also include experiences with transitional care interventions. Quantitative studies (i.e., surveys) were excluded as qualitative studies provide more in-depth information. The studies we analyzed in the current paper focused exclusively on the experiences of patients, family members, or healthcare professionals with multidisciplinary transitional care interventions, as this could provide insight into facilitators and barriers associated with their implementation.

### 2.1 Study selection

Briefly, the previously published meta-synthesis (van Grootel et al., 2024) identified studies through electronic searches in Medline, Embase, and Cinahl from inception until June 2024 that: (1) applied qualitative data collection methods (semi-structured interviews or focus groups), (2) included adult patients regardless of their diagnoses, family members, or healthcare professionals, (3) focused on experiences of hospital-to-home transitions. Studies investigating transitional care interventions that involved only one healthcare discipline (e.g., telephone follow-up by a nurse practitioner) were excluded, as were studies reporting on patients with a mental illness only or those requiring palliative care. For the present review, papers that did not focus on evaluating a multidisciplinary transitional care intervention but on "usual care" hospital-to-home transitions were also excluded.

### 2.2 Quality assessment of the included studies

Each article was independently assessed by two researchers (JvG and RC) using the Critical Appraisal Skills Programme checklist for qualitative research (CASP, 2018), following recommendations of Butler et al. (2016) (Supplementary material, Appendix 3). Articles without a statement on ethics (item nr. 7 = 0.0), and those with a sum score < 6.0 were excluded (Butler et al., 2016). Lastly, the papers were categorized into high-quality (score = 9.0–10.0), moderate-quality (score = 7.5–9.0), and fair-quality (score = 6.0–7.5). For full details on the study's search strategy, study selection procedure, quality assessment, and data extraction, we refer to the previous publication (van Grootel et al., 2024).

### 2.3 Theoretical framework

The Updated Consolidated Framework for Implementation Research is a widely used framework for identifying facilitators and barriers for the successful implementation of healthcare interventions (Damschroder et al., 2022). It comprises 39 subdomains associated with practical implementation organized into five domains: *Innovation* (i.e., the intervention being implemented), *Inner Setting* (i.e., the setting in which the intervention is implemented), *Outer Setting* (i.e., the setting in which the inner setting exists such as the healthcare system), *Individuals* (i.e., roles and characteristics of the stakeholders), and *Implementation Process* (i.e., activities and strategies used to implement the intervention). Applying this framework allows us to compare studies conducted in different settings and synthesize factors influencing intervention implementation. It also helps us understand which subdomains promote or inhibit health intervention adoption, implementation, and maintenance (Powell et al., 2017).

### 2.4 Data synthesis and analysis

We used the Updated Consolidated Framework for Implementation Research to guide the analysis and synthesis of facilitators and barriers for implementation reported in the studies. JvG and RC thoroughly read all the papers included. All studies were imported into MAXQDA 2022 (VERBI Software, 2021). JvG and RC independently coded all qualitative studies using a combination of inductive and deductive approaches. First, open codes were inductively generated by examining first-order constructs (participants’ direct quotes) and second-order constructs (study authors’ interpretations) relevant to the aim of the current review. JvG and RC compared these codes to ensure consistency. Once the final list of codes was constructed, the two coders deductively linked each code to a subdomain of the Consolidated Framework for Implementation Research framework (Damschroder et al., 2009). JvG and RC met regularly throughout the analysis to compare their results. Any disagreement was discussed until consensus was reached. The analysis results were discussed during reflexivity meetings involving the entire research team. Facilitators and barriers were identified and summarized by the five domains of the Consolidated Framework for Implementation Research. Then, all factors were organized into three categories:

1. General factors, i.e. factors relevant to the entire hospital-to-home transition,
2. Pre-discharge factors, i.e. factors relevant only to the hospital phase,
3. Post-discharge factors, i.e. factors relevant only to the home phase.

Finally, results were narratively described and summarized using Tables and Figures.

## 3 Results

### 3.1 Study selection and quality appraisal

The study selection process and quality appraisal results are described in detail in another publication (van Grootel et al., 2024). The PRISMA flowchart of the previous publication is provided in the supplementary material Appendix 1. Of the 53 articles included in the previous meta-synthesis, 12 evaluated patients', family members', or healthcare professionals' experiences with multidisciplinary transitional care interventions and hence were included. Two studies (Maximos et al., 2024; Kokorelias et al., 2023) were considered of high quality, six studies (Allen et al., 2020; Jepma et al., 2021; Allen et al., 2022; Cobley et al., 2013; Gustafsson et al., 2014; Lou et al., 2017) were of moderate quality, and four studies(Harvey et al., 2016; Major et al., 2021; Prinjha et al., 2009; Verweij et al., 2021) were of fair quality (Supplementary material, Appendix 2).

### 3.2 Study and population characteristics

The studies were published between 2013 and 2024 in five countries: Australia (n = 4), the Netherlands (n = 3), Canada (n = 2), the United Kingdom (n = 2), and Denmark (n = 1). Six studies investigated the perspectives of patients and/or family members, five explored those of healthcare professionals, and one combined the perspectives of patients, family members, and healthcare professionals. Patient populations were older (n = 7), stroke (n = 3), and former intensive care unit patients (n = 2) (Table 1).

**Table 1:** Characteristics of the included studies.

Author, year, country	Aim	Patient population	Professionals delivering the intervention	Work setting involved	Demographics N [Mean age (range), M/F]	Design and data collection method
Maximos et al., 2024, Canada	To examine the perspectives of healthcare professionals on a community-based, slow-stream rehabilitation, hospital-to-home transition program	Older people	Support/administrative staff , not further specified; nurse practitioner; physiotherapist; coordinator	Hospital and primary care	Healthcare professionals: 23[NA(NA), NA]	Semi-structured interviews and focus groups
Kokorelias et al., 2023, Canada	To report older adults' and family members’ experiences of receiving services from a hospital-to-home patient navigation program.	Older people	Not specified	Hospital and primary care	Patients: 9[79(68–95), 4/5] Family members: 5[60(50–73)2/3]	Qualitative descriptive study using semi-structured interviews
Allen et al., 2022, Australia	To co-design a communication tool to guide conversations about transitional care needs between healthcare practitioners and patients returning to the community	Older people with chronic conditions	Nurse practitioner Social worker Physician Physiotherapist Occupational therapist Pharmacist Case manager	Primary care and hospital	Healthcare professionals: 22 [NA (NA), 5/17]	Action research; semi structured interviews and focus groups
Allen et al., 2020, Australia	To evaluate healthcare practitioners’ perceptions of the feasibility and acceptability of a communication tool during transition from hospital to home	Older people with chronic conditions	Nurse practitioner; physiotherapist; social worker; occupational therapist; Pharmacist	Primary care and hospital	Healthcare professionals: 22 [NA (20–70+), NA]	Exploratory descriptive qualitative design; semi-structured interviews
Jepma et al., 2021, The Netherlands	To explore the experiences of participants who received a nurse-coordinated transitional care intervention	Older people with heart conditions	Nurse practitioner; physiotherapist	Primary care and hospital	Patients: 16[82 (71–89), 8/8]	Generic qualitative approach, not further specified; semi-structured interviews
Major et al., 2021, The Netherlands	To investigate the feasibility of an interdisciplinary rehabilitation program designed for patients with Post Intensive Care Syndrome transitioning from hospital to home	Former ICU patients	Physiotherapist; dietician; occupational therapist	Primary care	Healthcare professionals: 11[NA(NA), NA]	Mixed method, non-randomized, prospective feasibility study; focus group
Verweij et al., 2021, The Netherlands	To evaluate healthcare professionals' perspectives on the Cardiac Care Bridge nurse-coordinated transitional care intervention	Older people with heart conditions	Nurse practitioner; physiotherapist	Primary care, and hospital	Healthcare professionals: 19[41 (23–62), 2/17]	Mixed methods process evaluation; semi-structured interviews
Harvey et al., 2017, Australia	To describe the care transition experiences of people transitioning from hospital to home who followed a Geriatric Evaluation and Management model and improve that model	Older people	Nurse practitioner; occupational therapist; social worker; physiotherapist; physician; coordinator, not further specified	Primary care and hospital	Patients: 20 [82 (64–95), 12/7] Family members: 19 [NA (NA), NA] Healthcare professionals: 23[NA (NA), NA]	Exploratory, longitudinal case study; semi-structured interviews and focus groups
Lou et al., 2016, Denmark	To investigate how mild stroke patients and their partners experience and manage everyday life in a context of early supported discharge	Stroke	Nurse practitioner; physiotherapist; occupational therapist	Primary care and hospital	Patients: 22 [69 (41–85), 15/22]	Qualitative approach, not further specified; semi-structured interviews
Cobley et al., 2013, UK	To investigate patients' and carers' experiences of early supported discharge services	Stroke	Multidisciplinary specialist rehabilitation, not further specified	Primary care and hospital	Patients 27 [70 (NA), NA] Family members 15 [73 (NA), 2/13]	Thematic analysis; semi-structured interviews
Gustafsson et al., 2013, Australia	To investigate the experiences of people with stroke, during their transition from hospital to home, after participating in an inpatient outreach program	Stroke	Occupational therapist; physiotherapist; speech and language therapist	Primary care	Patients: 7 [61 (36–80), 3/4]	Qualitative approach, not further specified; semi-structured interviews
Prinjha et al., 2009, UK	To explores patients' perceptions and experiences of post ICU follow-up services	Former ICU patients	Nurse practitioner; physiotherapist; occupational therapist; physician; psychologist	Primary care and hospital	Patients: 34[52.1(23–76), 20/14]	Qualitative approach, not further specified; semi-structured interviews

Abbreviations: N= number, M= male, F= female, NA= not available, COPD= chronic obstructive pulmonary disease, ICU= intensive care unit

### 3.3 Interventions characteristics

The interventions comprised two to seven components, the most common being a comprehensive discharge needs assessment (n = 8), a discharge plan communicated to community care providers (n = 4) (pre-discharge), and follow-up home visits (n = 6) (Table 3). All interventions involved multidisciplinary teams comprising medical, nursing, or allied health professionals from hospitals and primary care (n = 10) or primary care only (n = 2) (Table 1).

### 3.4 Facilitators and barriers to implementation

**Fig. 1:**
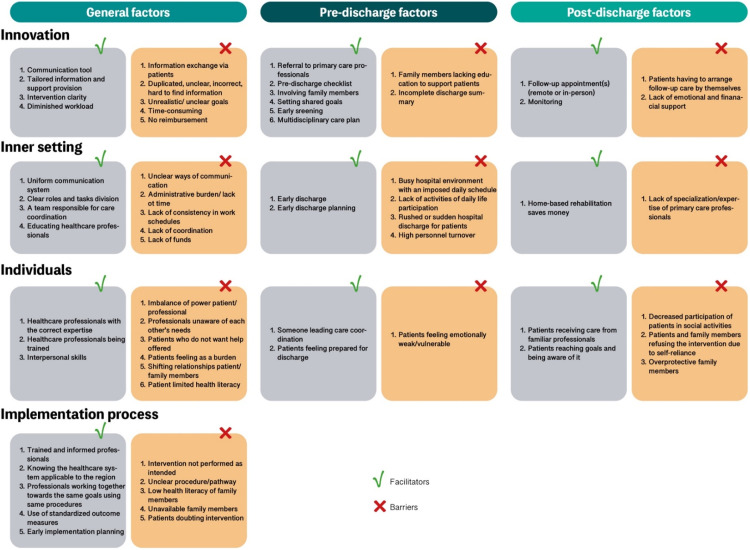
Facilitators and barriers per discharge phase.

We identified 79 factors influencing the implementation of multidisciplinary transitional care interventions. Table 2 provides an overview of the domains and subdomains of the Consolidated Framework for Implementation Research for which we identified these factors. Fig. 1 summarizes all factors organized per phase of transition. Table 4 provides quotes from the studies to illustrate the domains and subdomains of the Consolidated Framework for Implementation Research.

**Table 2:** Detailed description of identified facilitators and barriers.

Facilitators	Barriers
*1.Innovation*	
*Relative advantage*	
F1.1 A follow-up appointment after discharge (phone call/visit at home/outpatient clinic) F1.2 Referral to relevant primary care (allied health) professionals, ideally member of a specialized (allied) healthcare network F1.3 A communication tool can support information exchange between and within settings and healthcare professional-patient interaction F1.4 Use of a pre-discharge checklist for healthcare professionals	B1.1 Patients having to arrange follow-up care by themselves B1.2 Information exchange via patients is unsafe/unreliable
*Adaptability*	
F1.5 Tailoring information provision (appropriate quantity, timing, manner and format) F1.6 Tailoring the intervention according to individual patient goals (finding the right dose, frequency, and intensity not to overload patients)	B1.3 Duplicated, unclear, incorrect, or hard to find information
*Complexity*	
F1.7 Involving family members from day one (in transitional care decision making) F1.8 Setting shared goals for patients and family members F1.9 Early identification of patients with high-risk of requiring follow-up (allied) healthcare F1.10 Care plan developed by a multidisciplinary (allied) healthcare team addressing patients’ medical, nursing, psychosocial, and allied healthcare needs F1.11 Communication tool supports multidisciplinary collaboration between and within settings F1.12 Patients discharged early if they are properly monitored once home and if follow-up care in the community is coordinated	B1.4 Family members need to be educated to support patients B1.5 Lack of emotional and financial support after discharge
*Design*	
F1.13 Easy to understand transitional care intervention, giving clear expectations to patients, family members, and (allied health) professionals	B1.8 Not getting what was promised in the transitional care intervention B1.9 Unclear goals of the transitional care intervention
*Cost*	
F1.14 The transitional care intervention is diminishing workload	B1.10 Costs affect continuation of therapy B1.11 Time-consuming transitional care intervention B1.12 Insurance issues B1.13 Costs were reimbursed by the study (subsidy)
*2.Inner Setting*	
*Structural characteristics*	
F2.1 Being discharged home early enhances recovery and increases adherence to prescribed therapy F2.2 A unique communication system (for both hospital and primary care professionals)	B2.1 Busy hospital environment with an imposed daily schedule B2.2 Lack of activities of daily life participation B2.3 Communication through electronic patient record is not easy B2.4 Administrative burden of the transitional care intervention
*Relational connections*	
F2.3 Clear roles and tasks division between healthcare professionals F2.4 A team responsible for coordination within and between settings with a problem-solving approach	B2.5 Not having a leader to coordinate the transitional care intervention B2.6 Lack of consistency in work schedules between professionals of different settings B2.7 Lack of coordination between disconnected healthcare providers
*Communications*	
	B2.8 Unclear ways to communicate/who to contact for healthcare professionals
*Relative priority*	
F2.5 Early planning of discharge and assistance with arranging home equipment	B2.9 Rushed or sudden hospital discharge for patients
*Available resources*	
F2.6 Home based therapy can save money F2.7 Allowing enough time for educating healthcare professionals on providing transitional care interventions	B2.10 Healthcare professionals do not have the time for transitional care B2.11 High personnel turnover B2.12 The organization must be able to invest enough money into transitional care B2.13 Patients rushed out of hospitals due to high beds demand B2.14 Lack of specialization/expertise of primary care professionals
*3.Outer Setting*	
–	–
*4.Individuals Involved*	
Opinion leaders	
F4.1 Someone in the lead who coordinates the transition from hospital to home	
*Innovation deliverers*	
F4.2 Healthcare professionals with the correct expertise to adequately support patients during hospital-to-home transitions	B4.1 Imbalance of power between patient and healthcare professional B4.2 Healthcare professionals unaware of each other's needs
*Innovation recipients*	
	B4.4 Patients who do not want/need help offered through transitional care interventions
*Characteristics (need, capability, opportunity, motivation)*	
F4.3 Seeing and interacting with patients as normal persons F4.4 Healthcare professionals have the necessary interpersonal skills to include families F4.5 Patients feeling prepared for discharge F4.6 Patients who feel able to take back control over their lives after discharge F4.7 Patients prefer receiving care from a familiar primary care professional F4.8 Healthcare professionals are enthusiastic about learning new things F4.9 Patients reaching goals become more confident and remain motivated	B4.5 Patients feeling as a burden for their loved ones B4.6 Decreased participation of patients in social activities B4.7 Learning how to care is challenging for low-literate family members B4.8 Hard to adapt for patients when they are at home B4.9 Patients who do not want to go to the hospital again/do not wish receiving help B4.10 Families not having time for themselves B4.11 Low-literate patients require more time and attention B4.12 Overprotective family members B4.13 Shifting relationships between patients and family members during transitions B4.14 Patients feeling emotionally weak/vulnerable
*5.Implementation Process*	
*Planning*	
F5.1 Implementation should be planned early to fit healthcare professionals’ and patients’ needs and expectations	B5.1 Low adherence
*Tailoring strategies*	
F5.2 Healthcare professionals should be trained and informed F5.3 Knowing the healthcare system applicable to the region	B5.2 Unclear procedure/pathway for optimal hospital-to –home transitions
*Engaging*	
F5.4 Healthcare professionals working together towards the same goals, following the same guideline	B5.3 Transitional care intervention not performed as intended B5.4 Involving low-literate carers is challenging for professionals B5.5 Family members are not always available B5.6 Patients having doubts about the transitional care intervention
*Reflecting and evaluating*	
F5.5 Use of standardized outcome measures to monitor the impact of transitional care interventions

**Table 3:** Multidisciplinary transitional care interventions’ components.

Author, year	Pre-discharge intervention components	Post-discharge intervention components
Allen et al., 2020	‐ Improved professional patient communication in-hospital by means of a checklist ‐ Discharge planning needs assessment ‐ Self-management ‐ Family involvement	‐ Self-management
Allen et al., 2022	‐ Improved professional patient communication in-hospital by means of a checklist ‐ Discharge planning needs assessment ‐ Self-management ‐ Family involvement	‐ Self-management
Cobley et al., 2013	‐ Accelerated hospital discharge	‐ Home visits
Gustafsson et al., 2013		‐ Home visits ‐ Outpatient service
Harvey et al., 2017	‐ Discharge planning needs assessment ‐ Multidisciplinary discharge care plan	
Jepma et al., 2021	‐ Case management ‐ Discharge planning needs assessment ‐ Multidisciplinary discharge care plan ‐ Discharge plan communicated to community care providers	‐ Case management ‐ Home visits ‐ Continuous needs assessment and evaluation of the care plan after discharge ‐ Medication reconciliation
Kokorelias et al., 2023		‐ Case management ‐ Monitoring via follow-up appointments
Lou et al., 2016	‐ Discharge planning needs assessment ‐ Multidisciplinary discharge care plan ‐ Discharge plan communicated to community care providers	‐ Home visits ‐ Post-discharge needs assessment and evaluation of the care plan
Major et al., 2021	‐ Discharge planning needs assessment ‐ Discharge plan communicated to community care providers	‐ Home visits
Maximos et al., 2024	‐ Case management	‐ Outpatient service ‐ Self-management
Prinjha et al., 2009	‐ Discharge planning needs assessment	‐ Monitoring via follow-up appointments
Verweij et al., 2021	‐ Case management ‐ Discharge planning needs assessment ‐ Multidisciplinary discharge care plan ‐ Discharge plan communicated to community care providers	‐ Case management ‐ Home visits ‐ Continuous needs assessment and evaluation of the care plan after discharge ‐ Medication reconciliation

**Table 4:** Example participants’ quotes or authors’ interpretations.

**Innovation domain, 12 studies** (Allen et al., 2020; Allen et al., 2022; Cobley et al., 2013; Gustafsson et al., 2014; Harvey et al., 2016; Jepma et al., 2021; Lou et al., 2017; Major et al., 2021; Prinjha et al., 2009; Verweij et al., 2021; Maximos et al., 2024; Kokorelias et al., 2023)	
**Subdomain**	**Example participants’ quote and/or authors’ interpretation** (quality according to Critical Appraisal Skills Programme score)
Relative advantage	“[…] caregiver participants described how the navigator connected them with appropriate services to meet their informational needs and provided them with oppornmities to socialize. As one participant shared, *it wasn't just going to a caregiver support group to learn how to support mental health needs, but it was an oppornmity to meet friends and real people in similar situations*- Caregiver, Female, P4. Similarly, the navigators directed clients to appropriate services, assisted them with access those services, and supported their self-advocacy […] *she [navigator] told me to just try and continue trying, so I started. I almost demanded I needed the [Community Agency] services and guess what? I got them-* Older Adult, Female, P2.” (Kokorelias et al., 2023)(High quality)
Adaptability	“Many respondents described the information provided as failing to address their own needs and issues of concern: *You read the pamphlets, the leaflets and things, what to look for with strokes, but I mean the thing is, a lot of the things in there weren't applicable* (interview 12; patient)” (Cobley et al., 2013)(Moderate quality)
Complexity	“[…] the TRANSITION tool supported healthcare practitioners to know what to ask, which enabled holistic assessment information to be collected about discharge care needs, identification of problems and referral to allied health […] participants commented that they considered specific domains of the tool were valuable as a guide for “knowing what to ask.” These included questions in relation to memory, services available to patients at home, injury risk, medications, instruction and education, and if the patient had any other concerns about going home.” (Allen et al., 2020)(Moderate quality)
Design quality and packaging	“*I think [The program] is designed for people but a certain level of independence and ability to follow through on commands and able to benefit from the program.* (P11)” (Maximos et al., 2024)(High quality)
Cost	“*You can design an intervention program with a desired frequency and for a desired duration but with limited coverage, you run out really quickly. Treatment is so dependent on individual circumstances and that makes it difcult. This patient I have, for example I have let him come for 2 additional months without letting him ... paying it myself because he has unemployment benefts only and I thought it important to get him back to how he was before* (physiotherapist)” (Major et al., 2021)(Fair quality)
**Inner setting domain, 12 studies** (Allen et al., 2020; Allen et al., 2022; Cobley et al., 2013; Gustafsson et al., 2014; Harvey et al., 2016; Jepma et al., 2021; Lou et al., 2017; Major et al., 2021; Prinjha et al., 2009; Verweij et al., 2021; Maximos et al., 2024; Kokorelias et al., 2023)	
**Subdomain**	**Example participants’ quote and/or authors’ interpretation** (quality according to Critical Appraisal Skills Programme score)
Structural characteristics	“The couples reported that the home atmosphere was less stressful and that they were able to ask questions more freely. *It's just more relaxed... Like, at the hospital I sit in the chair, right? At the patient side of the table. But at home it's different. It's my home ground so the roles are a bit different. She's the visitor. That puts me more in control. In a way.* (Jakob, patient)” (Lou et al., 2017)(Moderate quality) “When they first came home, many had felt insecure about no longer being in the safe environment of the hospital.” (Prinjha et al., 2009)(Fair quality)
Relational connections	“Participants reported that the community nurse and physical therapist collaborated together and with other involved healthcare providers […] Patient: *Those medicines were changed several times(…).* Interviewer: *Was it difficult for you that they were changed so frequently?* Patient*: No, actually, but I do not know which medicines I should have then, then everything is just all let loose [in multi-dose drug dispenser].* Interviewer: *Okay and the community nurse helped with that, I understand?* Patient*: Yes, the hospital told her [community nurse] which ones had to get out.* (P3, male, 81 years)” (Jepma et al., 2021)(Moderate quality)
Communications	“[…] all interviewed healthcare professionals mentioned the value of the collaboration and integrated alternative communication routes such as contact by telephone: *I think we, the physical therapist and I, accomplished a lot. There was a woman, … She went for groceries with her walker the first day after discharge; and there she sat in the middle of the street. She simply overestimated her situation… Together with the physical therapist we enabled her to do the groceries again; then, you feel satisfied…* (Respondent 8 community nurse)”(Verweij et al., 2021)(Moderate quality)
Relative priority	There was a consensus of preference among participants [...] for returning to their home environment as soon as possible: *Well, I was glad I was going home … I'm not saying the hospital was horrible, but I just didn't like being in the hospital* (interview 14; patient)” (Cobley et al., 2013)(Moderate quality)
Available resources	“All participants reported that the tool took between 10 and 25 min to use and some participants noted that the tool would be more feasible if more concise”(Allen et al., 2022)(Moderate quality)
**Individuals domain, 12 studies** (Allen et al., 2020; Allen et al., 2022; Cobley et al., 2013; Gustafsson et al., 2014; Harvey et al., 2016; Jepma et al., 2021; Lou et al., 2017; Major et al., 2021; Prinjha et al., 2009; Verweij et al., 2021; Maximos et al., 2024; Kokorelias et al., 2023)	
**Subdomain**	**Example participants’ quote and/or authors’ interpretation** (quality according to Critical Appraisal Skills Programme score)
Opinion leaders	“Providers stressed the potential of the GP in care coordination to help navigate the service landscape […]: *[The general practitioner]...knows someone for 30 years... far better that person presents to their general practitioner to have things sorted out than they present to the emergency department*. (Community care provider)” (Harvey et al., 2016)(Fair quality)
Innovation deliverers	“The participants reported that the ESD teams were very knowledgeable and experienced with stroke rehabil-itation and that they were able to put themselves in the patient's shoes” (Lou et al., 2017)(Moderate quality) “However, participants had some difficulties to mention the specific role of the community care nurse which was primary to recognize health deterioration early.” (Jepma et al., 2021)(Moderate quality)
Characteristics	“Look, I can do all those exercises, and, in the begin-ning, you were uhm well then you really had to catch up. But now I just recover in a minute, two minutes and then it is back to normal. So, then you see, you feel that you are building up something and that is important. (Patient 5, male, 76 years)” (Jepma et al., 2021)(moderate quality) “A few participants alluded to not being interested in a patient navigation program. Some of these older adults believed that they did not need patient navigation because they *are always able to make do alone at home* following a hospitalization-Older Adult, Male, P8.” (Kokorelias et al., 2023)(High quality) “While four clients demonstrated an eagerness for re-engaging with their lives and taking control, there were three clients who expressed a sense of grief in regards to their new lives […]: *Sitting here, or sitting out there on the veranda, or going for a ride in the car over to [shopping centre]; something like that. I just sit there, fairly quiet, very quiet. Very sad, very sad.* […] These clients were less likely to describe the benefits of STRENGTH and reported experiencing a much more sedentary lifestyle, reduced socialization, increased dependence on others for basic self-cares, and reduced participation in meaningful activities.” (Gustafsson et al., 2014)(Moderate quality)
**Implementation process domain, 6 studies** (Cobley et al., 2013; Jepma et al., 2021; Major et al., 2021; Prinjha et al., 2009; Verweij et al., 2021; Maximos et al., 2024)	
**Subdomain**	**Example participants’ quote and/or authors’ interpretation** (quality according to Critical Appraisal Skills Programme score)
Assessing needs	“Successful implementation of the tool will require a carefully planned strategy to facilitate trans-lation of the tool into the routine practice of ward-based nurses’ and support their roles in communication and assessment during older patients’ care transitions” (Allen et al., 2020)(Moderate quality)
Tailoring strategies	“ Implementation of a communication instrument, such as the TRANSITION tool, into practice by ward-based nurses will require consideration of existing practices in order to streamline care pro-cesses and minimise service duplication.” (Allen et al., 2020)(Moderate quality)
Engaging	“The collaboration between physical therapists and community nurses was considered valuable to motivate patients when working on the same goals from different perspectives.” (Verweij et al., 2021)(Fair quality)
Reflecting and evaluating	“Our findings stress the importance of using carer, in addition to patient, outcome mea-sures for the purposes of evaluating the effective-ness of a rehabilitation service and emphasize the inadequacy of using only traditional patient-centred outcome measures” (Cobley et al., 2013)(Moderate quality) “[…]the implementation of standardized measures in a model of care would be beneficial for communicating patient progress and needs among different caresettings, as well as would providing evidence to support the need for the program, to sustain current funding levels, and to advocate for increased expansion and funding in the future.” (Maximos et al., 2024)(High quality)

### 3.5 Innovation

Facilitators and barriers from all studies (two high, six moderate, and four fair-quality) were mapped under five subdomains: ***relative advantage, adaptability, complexity, design quality and packaging***, and ***cost*** (Allen et al., 2020; Allen et al., 2022; Cobley et al., 2013; Gustafsson et al., 2014; Harvey et al., 2016; Jepma et al., 2021; Lou et al., 2017; Major et al., 2021; Prinjha et al., 2009; Verweij et al., 2021; Maximos et al., 2024; Kokorelias et al., 2023).

### 3.6 General factors

Several factors were found to be relevant throughout the entire hospital-to-home transition. The interventions enabled tailored information provision (***adaptability***) regarding quantity, clarity, correctness, and timing(Allen et al., 2020; Cobley et al., 2013; Harvey et al., 2016; Jepma et al., 2021; Lou et al., 2017; Prinjha et al., 2009; Maximos et al., 2024; Kokorelias et al., 2023). They also facilitated personalized and multidisciplinary support provision to help patients achieve their goals by addressing their medical, nursing, psychosocial, and rehabilitation needs(Allen et al., 2020; Allen et al., 2022; Gustafsson et al., 2014; Harvey et al., 2016; Major et al., 2021; Prinjha et al., 2009; Verweij et al., 2021; Maximos et al., 2024; Kokorelias et al., 2023). However, the ***design*** and ***cost*** of transitional care interventions often threatened implementation success. To ensure adherence and maintain the interventions' ***complexity***, the interventions and their objectives had to be clear to patients, family members, and healthcare professionals(Allen et al., 2020; Cobley et al., 2013; Gustafsson et al., 2014; Lou et al., 2017; Major et al., 2021; Maximos et al., 2024; Kokorelias et al., 2023). In addition, time-consuming interventions threatened implementation success, especially when health insurance did not cover expenses related to the time spent by healthcare professionals to coordinate care across settings (Major et al., 2021; Verweij et al., 2021).

### 3.7 Pre-discharge factors

In the pre-discharge phase, multidisciplinary transitional care interventions had the ***advantage*** that they could help identify patients likely to require follow-up (allied health)care after hospital discharge (Allen et al., 2022; Harvey et al., 2016; Jepma et al., 2021; Major et al., 2021; Kokorelias et al., 2023). The interventions ensured that a comprehensive care plan was designed to address patients' medical, nursing, and allied healthcare needs and goals (***complexity***) (Gustafsson et al., 2014; Harvey et al., 2016; Major et al., 2021; Maximos et al., 2024; Kokorelias et al., 2023). The referral of patients to the appropriate healthcare professionals in the community was also facilitated, and family members were involved and guided to support their loved ones after hospital discharge (***complexity, relative advantages***) (Jepma et al., 2021; Prinjha et al., 2009; Maximos et al., 2024; Kokorelias et al., 2023). Family members' involvement consisted, for example, of education on how to support patients in their physical and emotional recovery and how to ***adapt*** their daily routines, everyday activities, and relationships to reduce the burden of recovery (Cobley et al., 2013; Harvey et al., 2016; Jepma et al., 2021; Lou et al., 2017; Maximos et al., 2024; Kokorelias et al., 2023).

### 3.8 Post-discharge factors

After discharge from the hospital, multidisciplinary transitional care interventions facilitated effective patient-professional communication and information exchange between healthcare professionals across settings. This ensured patients received follow-up care immediately after discharge (***relative advantages***) (Allen et al., 2022; Cobley et al., 2013; Jepma et al., 2021; Lou et al., 2017; Major et al., 2021; Prinjha et al., 2009; Verweij et al., 2021; Kokorelias et al., 2023).

### 3.9 Inner setting

Facilitators and barriers from all studies (two high, six moderate, and four fair-quality) were linked to five subdomains: ***structural characteristics, relational connections, communications, relative priority, and available resources***(Allen et al., 2020; Allen et al., 2022; Cobley et al., 2013; Gustafsson et al., 2014; Harvey et al., 2016; Jepma et al., 2021; Lou et al., 2017; Major et al., 2021; Prinjha et al., 2009; Verweij et al., 2021; Maximos et al., 2024; Kokorelias et al., 2023).

### 3.10 General factors

Although transitional care interventions offered a more efficient work structure and facilitated communication between professionals, several barriers threatened their viability pre- and post-discharge. In some cases, such interventions increased the administrative burden (***available resources***), which healthcare professionals perceived as detrimental since they had limited time to begin with(Allen et al., 2020; Allen et al., 2022; Harvey et al., 2016; Verweij et al., 2021). In addition, digital ***communication*** tools were not easy for everyone, and some healthcare professionals lacked sufficient training to use these tools optimally, which hindered the execution of the interventions (Cobley et al., 2013; Harvey et al., 2016; Verweij et al., 2021; Maximos et al., 2024).

### 3.11 Pre-discharge factors

Some interventions included a digital ***communication*** platform (structural characteristics) or protocols detailing how and when information should be exchanged between professionals (e.g., during multidisciplinary meetings or individual appointments) (Allen et al., 2020; Allen et al., 2022; Harvey et al., 2016; Jepma et al., 2021; Verweij et al., 2021; Maximos et al., 2024). This resulted in better discharge planning, sometimes even allowing patients to be discharged earlier (***relative priority***) to continue their recovery at home (Cobley et al., 2013; Lou et al., 2017; Prinjha et al., 2009; Verweij et al., 2021; Kokorelias et al., 2023). Early discharge benefited many patients who preferred the safe environment of their home to recover while they reduced the shortage of hospital beds and related costs (***relative priority and available resources***) (Cobley et al., 2013; Gustafsson et al., 2014; Jepma et al., 2021; Lou et al., 2017; Major et al., 2021; Prinjha et al., 2009; Verweij et al., 2021; Maximos et al., 2024).

### 3.12 Post-discharge factors

One condition for early discharge to benefit patients and reduce costs was that follow-up care would be available immediately after discharge. However, in some cases, this was impossible due to a lack of expertise of the primary care professionals patients were referred to (***available resources***) (Prinjha et al., 2009; Maximos et al., 2024).

### 3.13 Individuals

Facilitators and barriers from all studies (two high, six moderate, and four fair-quality) were linked to four subdomains: ***opinion leaders, innovation deliverers, innovation recipients***, and ***characteristics*** (Allen et al., 2020; Allen et al., 2022; Cobley et al., 2013; Gustafsson et al., 2014; Harvey et al., 2016; Jepma et al., 2021; Lou et al., 2017; Major et al., 2021; Prinjha et al., 2009; Verweij et al., 2021; Maximos et al., 2024; Kokorelias et al., 2023).

### 3.14 General factors

Healthcare professionals (***deliverers***) who possessed good interpersonal skills and were trained to consider the preferences of patients and their families regarding the type and quantity of support they required (***characteristics***) resulted in positive experiences for patients and family members(Allen et al., 2020; Cobley et al., 2013; Gustafsson et al., 2014; Harvey et al., 2016; Jepma et al., 2021; Lou et al., 2017; Major et al., 2021; Prinjha et al., 2009; Maximos et al., 2024; Kokorelias et al., 2023). On the one hand, patients valued being treated in a personable manner and interacting with their healthcare providers, who motivated them and gave them confidence in their ability to recover fully. On the other hand, the continuous training of healthcare professionals in delivering the interventions kept them motivated as they appreciated developing their skills to provide the best care (Cobley et al., 2013; Gustafsson et al., 2014; Harvey et al., 2016; Jepma et al., 2021; Lou et al., 2017; Major et al., 2021; Prinjha et al., 2009; Verweij et al., 2021).

In some cases, healthcare professionals failed to provide adequate support, which hindered the execution of the interventions (Cobley et al., 2013; Gustafsson et al., 2014; Harvey et al., 2016; Jepma et al., 2021; Lou et al., 2017; Major et al., 2021; Prinjha et al., 2009). For example, patients with a lower socio-economic status sometimes felt lost when trying to navigate community services because they lacked self-management skills (***recipients’ characteristics***). Furthermore, healthcare professionals (***deliverers***) were sometimes unaware of each other's roles, which was one reason they failed to provide tailored support (Allen et al., 2020; Allen et al., 2022; Jepma et al., 2021).

### 3.15 Pre-discharge factors

During the hospital phase, having a trained or experienced case manager (***opinion leader***) coordinating care who was familiar with patients and aware of the involved healthcare professionals' roles and tasks facilitated the interventions' success (Allen et al., 2020; Harvey et al., 2016; Jepma et al., 2021; Lou et al., 2017; Prinjha et al., 2009; Maximos et al., 2024; Kokorelias et al., 2023).

### 3.16 Post-discharge factors

During the home phase, several ***characteristics*** of patients and family members (***recipients***) facilitated or hindered intervention success. Participating in the intervention and witnessing positive outcomes in their physical, cognitive, and communication abilities motivated patients and family members to adhere to it (Cobley et al., 2013; Gustafsson et al., 2014; Lou et al., 2017; Major et al., 2021; Maximos et al., 2024). However, when patients had a more sedentary lifestyle and reduced level of participation in meaningful (social) activities, they did not see the benefits of the intervention, which, in turn, hindered their adherence to it (Gustafsson et al., 2014). Furthermore, some patients and family members refused the intervention because they believed they had enough tools to self-manage recovery (Gustafsson et al., 2014; Harvey et al., 2016; Jepma et al., 2021; Kokorelias et al., 2023).

### 3.17 Implementation process

Facilitators and barriers from six studies (one high, two moderate, and three fair-quality) were linked to ***planning, tailoring strategies, engaging***, and r***eflecting and evaluating*** (Cobley et al., 2013; Jepma et al., 2021; Major et al., 2021; Prinjha et al., 2009; Verweij et al., 2021; Maximos et al., 2024)

### 3.18 General factors

Standardized outcome measures facilitated monitoring the interventions' impact, thus indicating if adaptation was needed (***reflecting and evaluating***) (Cobley et al., 2013; Prinjha et al., 2009; Maximos et al., 2024). In addition, understanding the local context, for example, to determine which professionals are best suited to deliver the interventions and how to integrate this with their other tasks facilitated implementation (***tailoring strategies***) (Allen et al., 2020).

However, several factors hindered successful implementation. In some cases, the interventions were not performed as intended because procedures and protocols were unclear, or there was low adherence because some patients were unaware of the interventions' objectives ***(planning, engaging***). In addition, it was often challenging to involve family members as they were unavailable at the same time as healthcare providers (***engaging***) (e.g., because they were working) (Jepma et al., 2021; Verweij et al., 2021).

## 4 Discussion

This qualitative review used the Updated Consolidated Framework for Implementation Research to synthesize healthcare professionals', patients', and family members' views on and experiences with multidisciplinary transitional care interventions. It provides insight into potential facilitators and barriers to implementing such interventions. In total, 79 facilitators and barriers were identified. Overall, most facilitators and barriers identified (88 %), supported by high-quality evidence, were mapped across three domains of the CFIR: (1) ***innovation***: ***relative advantage, adaptability, complexity, design quality and packaging, and cost***; (2) ***Inner setting: structural characteristics, relational connections, and communications***; (3) ***Individuals involved: opinion leaders, innovation deliverers, innovation recipients, and characteristics***. In contrast to these three domains, ***the Outer setting*** and ***Implementation process*** domains contained no or a limited number of factors. The main facilitators to implementing multidisciplinary transitional care interventions included "identification of patients' follow-up care needs" and "effective referral to the right professionals" (pre-discharge), "tailored follow-up care in the community" (post-discharge), and "effective communication between stakeholders across settings" (general). The main barriers were "shortage of hospital beds" and "lack of time" (pre-discharge), "lack of available allied healthcare professionals in the community" (post-discharge), and "inconsistencies between stakeholders' schedules" and "intervention costs" (general).

### 4.1 Innovation

Although this systematic review is the first one to provide a comprehensive overview of factors for the successful implementation of multidisciplinary transitional care interventions, previous qualitative reviews have examined the experiences and needs of patients, family members, and healthcare professionals during hospital-to-home transitions for chronically ill patients, stroke survivors, and patients requiring allied healthcare after discharge (Chen et al., 2021; Sun et al., 2023; van Grootel et al., 2024). According to these papers, patients and their families often felt that they were not prepared for discharge during hospitalization, which resulted in them feeling lost once they were home and/or being unable to manage their recovery. Post-discharge, patients were heavily dependent on their family members for their care. At times, family members were unsure how to assist their loved ones with managing their symptoms and performing their daily activities and would have appreciated receiving more support in this. Both patients and family members regretted the lack of follow-up services. Within the ***innovation domain*** of the implementation framework, the subdomains ***relative advantage, adaptability,*** and ***complexity*** highlighted that multidisciplinary transitional care interventions could offer a viable solution to address those needs as they facilitate the provision of tailored support and information and the involvement of family members throughout the hospital-to-home transition. Most factors identified within the ***innovation domain*** were mapped under ***complexity***, meaning multidisciplinary transitional care interventions can be complex and include a wide range of components (e.g., family involvement, multidisciplinary care plan, or communication between hospitals and primary care settings). In a recent comprehensive meta-analysis, Tyler et al. investigated whether the effectiveness of mono- and multidisciplinary transitional care interventions differed depending on the number of components they contained (Tyler et al., 2023). They found that interventions with fewer components were more effective in reducing readmissions and mortality than more complex ones. A potential reason could be that simpler interventions may be easier to implement and maintain.

On the other hand, we found that multidisciplinary transitional care interventions did not always address stakeholders' needs and expectations, because of barriers related to the ***design quality and packaging*** and ***cost*** subdomains. Regarding ***design quality and packaging***, the interventions’ objectives were not always clear to patients, family members, or healthcare professionals and were sometimes time-consuming, threatening their sustainability throughout the entire hospital-to-home transition. Hence, ensuring the intervention is ‘simple enough’, clear, and accessible to all stakeholders and continuously evaluating its processes and execution may promote adherence and sustainable maintenance.

In addition, intervention ***cost*** and reimbursement issues are challenging to address at the level of care settings. One of the most common healthcare provider payment methods is the fee-for-service model, in which healthcare providers are reimbursed for each service delivered (Bohler et al., 2024; Wagenschieber and Blunck, 2024). This model lacks incentives to promote quality rather than quantity of care. In recent years, alternative financing models have emerged, such as bundled payments consisting of one-off or periodic payments for various services to provide comprehensive care or value-based payments that reward providers who exceed their performance on quality and cost measures (Struijs et al., 2020; Zanotto et al., 2021). However, there is no conclusive evidence regarding the impact of such financing models for multidisciplinary transitional care interventions, and further research is needed to assess whether these would improve care quality and reduce costs.

### 4.2 Inner setting

Within the ***inner setting domain****,* we found that multidisciplinary transitional care interventions offered an opportunity to improve the work structure between professionals within the hospital (during the pre-discharge phase) and between those working within and outside the hospital (during the post-discharge phase). Previous qualitative reviews on patients’ and professionals’ experiences with hospital-to-home transitions identified that ineffective communication between professionals and a lack of standardized work processes hindered effective transitions (Sun et al., 2023; van Grootel et al., 2024). Professionals also indicated it was unclear whose role it was to coordinate follow-up care. In relation to this, we mapped several facilitators under the ***structural characteristics*** and ***relational connections*** subdomains. These factors highlighted that multidisciplinary transitional care interventions have the potential to address negative experiences regarding ineffective communication and lack of formal work processes, as they might provide standardized protocols and infrastructure to ensure effective collaboration and coordination between settings. However, the several barriers identified under ***structural characteristics, relational connections***, and ***available resources*** indicate that, in some cases, multidisciplinary transitional care interventions failed to address stakeholders’ needs and expectations during both the pre-and post-discharge phases. Such barriers include high personnel turnover, lack of consistency in work schedules between professionals across settings, or the administrative burden of the interventions. This implies that being fully aware of the situation (settings, healthcare professionals, patient populations involved and healthcare system) before implementing a multidisciplinary transitional care intervention is essential to developing realistic and practical solutions (i.e., "simple enough" interventions).

Furthermore, in recent years, technological tools and health information exchange platforms have been developed to support collaboration across healthcare settings and avoid the administrative burden of double registration (Genovese et al., 2022). These technological tools might be a viable alternative to compensate for staff shortages and facilitate communication between stakeholders. However, such tools are generally poorly implemented, and further research is needed to find solutions to integrate them at a systemic level.

### 4.3 Individuals

Within the ***Individuals domain****,* our findings indicate that during the pre-and post-discharge phases, the personal ***characteristics*** of healthcare professionals delivering multidisciplinary transitional care interventions, such as their experience, training, and interpersonal skills, can make patients feel valued and treated as persons and give them more confidence in their ability to recover fully. This finding aligns with a recent review that examined barriers and facilitators to implementing person-centered care in clinical practice (Grover et al., 2022). That review found healthcare professionals to play a pivotal role in implementing person-centered care through professional development through training or patient interaction. However, we showed that healthcare professionals sometimes failed to provide adequate support because they lacked expertise/specialization or when patients had lower socioeconomic status or literacy. Patients with lower health literacy levels may have a more limited capacity to self-manage their recovery. Extensive research has been conducted on interventions to improve health literacy in a wide range of patient populations (Walters et al., 2020). Such interventions consist of, for example, individual or group health counseling sessions, but could also be done through multimedia learning, text, or social media messages and were shown to improve patients’ knowledge, activation, self-efficacy, and behaviors (e.g., nutritional behaviors, physical activity). This implies that providing such interventions to patients with lower health literacy could improve their capacity to participate in multidisciplinary transitional care interventions and actively self-manage their conditions.Therefore, when designing future multidisciplinary transitional care interventions, additional efforts should be made to ensure patients are referred to professionals with the right expertise (e.g., through specialized primary care networks) and provide alternative solutions for patients with more limited self-management capacity.

### 4.4 Strengths and limitations

This systematic review is the first to provide insight into facilitators and barriers to implementing multidisciplinary transitional care interventions using the Consolidated Framework for Implementation Research, which can guide strategies for successful implementation. It included an extensive systematic search, a thorough qualitative synthesis using inductive and deductive approaches, and different patient populations. However, some limitations to our study can be identified. First, many researchers participated in the selection process, which may have negatively affected uniformity in article selection. We used sample checks to minimize this selection bias and held regular meetings throughout the screening process to ensure consistency. Second, only twelve articles were included, and we may not have reached thematic saturation. Therefore, readers wishing to implement multidisciplinary transitional care interventions should bear in mind that our list of factors is non-exhaustive and perform a thorough needs assessment of their specific situations. Third, the included studies were conducted in the European Union, Canada, the United Kingdom, and Australia. Therefore, the results cannot be generalised to contexts that are markedly different from these healthcare systems. Finally, we identified no or very few factors within the *outer setting* and *implementation process* domains, likely because the included studies only investigated user experiences (patients, family members, and healthcare professionals). The experiences and views of researchers or clinicians who developed and implemented the interventions might have provided more insight into these domains. However, such studies are lacking.

## 5 Conclusions

While individual qualitative studies provide insight into a limited number of factors related to the successful implementation of multidisciplinary transitional care interventions, this review provides a comprehensive overview of facilitators and barriers, which can serve as a non-exhaustive inventory list to inspire researchers and clinicians who wish to implement a multidisciplinary transitional care intervention in their settings. They indicate which components of the Consolidated Framework for Implementation Research require particular attention when designing implementation strategies. However, these facilitators and barriers may not all be relevant to specific situations. Therefore, performing a thorough needs assessment specific to the environments, settings, and populations the intervention targets is essential to drawing implementation strategies. Further research is needed to determine how to incorporate the factors identified into strategies for the successful implementation of multidisciplinary transitional care interventions.

## Data availability

Data will be available upon request via DataVerseNL. https://doi.org/10.34894/9HACDB

## Funding sources

This work was funded by 10.13039/501100001826ZonMw (Grants 10270022110008 and 10270022110004). The funding source had no role in any of the decisions taken in planning and conducting the project or publishing the results.

## CRediT authorship contribution statement

**Romain Collet:** Conceptualization, Data curation, Formal analysis, Investigation, Methodology, Project administration, Software, Validation, Visualization, Writing – original draft, Writing – review & editing. **Juul van Grootel:** Conceptualization, Data curation, Formal analysis, Investigation, Methodology, Project administration, Software, Validation, Visualization, Writing – original draft, Writing – review & editing. **Marike van der Leeden:** Conceptualization, Data curation, Methodology, Supervision, Validation, Writing – review & editing. **Marike van der Schaaf:** Conceptualization, Data curation, Funding acquisition, Resources, Writing – review & editing. **Johanna van Dongen:** Writing – review & editing, Data curation, Conceptualization. **Suzanne Wiertsema:** Writing – review & editing, Data curation, Conceptualization. **Edwin Geleijn:** Writing – review & editing, Data curation, Conceptualization. **Mel Major:** Writing – review & editing, Data curation, Conceptualization. **Raymond Ostelo:** Conceptualization, Data curation, Funding acquisition, Methodology, Resources, Supervision, Validation, Writing – review & editing.

## Declaration of competing interest

The authors declare the following financial interests/personal relationships which may be considered as potential competing interests: Marike van der Schaaf and Raymond Ostelo reports financial support was provided by Netherlands Organisation for Health Research and Development. If there are other authors, they declare that they have no known competing financial interests or personal relationships that could have appeared to influence the work reported in this paper.

## Appendix Supplementary materials

Image, application 1
