# Supplementary material for: Facilitators, barriers, and guidance to successful implementation of multidisciplinary transitional care interventions: A qualitative systematic review using the consolidated framework for implementation research
Source: Int J Nurs Stud Adv. 2024 Nov 29;8:100269. doi: 10.1016/j.ijnsa.2024.100269 (PMC11647461; doi:10.1016/j.ijnsa.2024.100269)
Supplement: Supplementary file 1 [file mmc1.docx]

Appendix 1: PRISMA Flowchart of the original meta-synthesis(van Grootel et al., 2024)


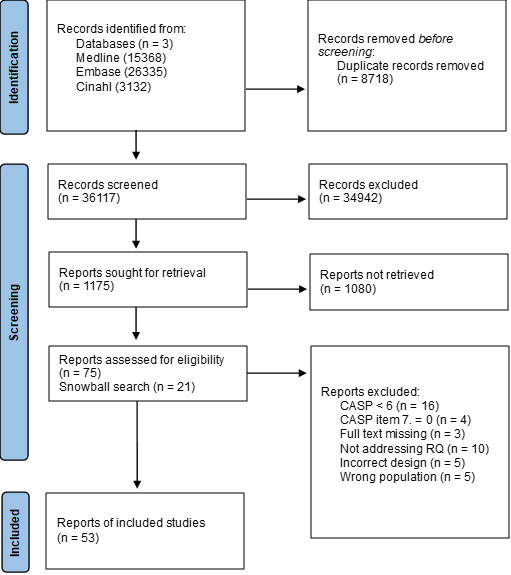


Abbreviation: CASP= Critical Appraisal Skills Program checklist for qualitative research

Appendix 2: Quality appraisal

| **First authors' last name** | **Year** | **CASP items** | | | | | | | | | | **Total score** | **Category** |
| --- | --- | --- | --- | --- | --- | --- | --- | --- | --- | --- | --- | --- | --- |
|  |  | 1 | 2 | 3 | 4 | 5 | 6 | 7 | 8 | 9 | 10 |  |  |
| Allen | 2020 | 1.0 | 1.0 | 0.5 | 0.5 | 1.0 | 0.5 | 1.0 | 0.0 | 1.0 | 1.0 | 7.5 | Moderate |
| Allen | 2022 | 1 | 1.0 | 1.0 | 0.5 | 1.0 | 0.5 | 1.0 | 0.5 | 1.0 | 1.0 | 8.5 | Moderate |
| Cobley | 2013 | 1.0 | 1.0 | 0.5 | 0.5 | 1.0 | 0.0 | 1.0 | 1.0 | 1.0 | 0.5 | 7.5 | Moderate |
| Gustafsson | 2013 | 1.0 | 0.5 | 1.0 | 1.0 | 0.5 | 0.0 | 1.0 | 0.5 | 1.0 | 1.0 | 7.5 | Moderate |
| Harvey | 2017 | 1.0 | 1.0 | 1.0 | 1.0 | 0.5 | 0.0 | 1.0 | 0.5 | 1.0 | 0.0 | 7 | Fair |
| Jepma | 2021 | 1.0 | 1.0 | 1.0 | 0.5 | 1.0 | 1.0 | 1.0 | 1.0 | 1.0 | 1.0 | 9.5 | Moderate |
| Kokorelias | 2023 | 1.0 | 1.0 | 1.0 | 1.0 | 1.0 | 1.0 | 1.0 | 1.0 | 1.0 | 1.0 | 10 | High |
| Lou | 2016 | 1.0 | 1.0 | 0.5 | 0.5 | 1.0 | 0.0 | 1.0 | 1.0 | 1.0 | 1.0 | 8 | Moderate |
| Major | 2021 | 1.0 | 1.0 | 0.5 | 1.0 | 0.0 | 0.0 | 1.0 | 0.0 | 1.0 | 0.5 | 6 | Fair |
| Maximos | 2024 | 1.0 | 1.0 | 1.0 | 1.0 | 1.0 | 0.5 | 1.0 | 1.0 | 1.0 | 0.5 | 9 | High |
| Prinjha | 2009 | 1.0 | 1.0 | 0.5 | 0.5 | 0.5 | 0.0 | 1.0 | 0.0 | 0.5 | 1.0 | 6 | Fair |
| Verweij | 2021 | 1.0 | 1.0 | 0.5 | 0.5 | 1.0 | 0.0 | 1.0 | 0.0 | 1.0 | 0.5 | 6.5 | Fair |
| Abbreviations: CASP Critical Appraisal Skills Program  1. Was there a clear statement of the aims of the research?  2. Is a qualitative methodology appropriate?  3. Was the research design appropriate to address the aims of the research?  4. Was the recruitment strategy appropriate to the aims of the research?  5. Was the data collected in a way that addressed the research issue?  6. Has the relationship between researcher and participants been adequately considered?  7. Have ethical issues been taken into consideration? | | | | | | | | | | | | | |

Appendix 3: Reviewer Guidelines for Using the CASP Checklist(Butler et al., 2016)

| Item | Guidelines |
| --- | --- |
| Question 2: Appropriate for qualitative methodology | Exclude if inappropriate |
| Question 3: Research design | *Yes*- Specifically states research design, with justification |
|  | *Unsure*- Outline of research design only |
|  | *No*- Not discussed or inappropriate to research question |
| Question 5: Data collection | *Yes*- Addresses 4 or more items listed on the CASP checklist |
|  | *Unsure*- Addresses 2–3 items listed on the CASP checklist |
|  | *No-* Addresses less than 2 items |
| Question 7: Ethical considerations | Exclude if unclear or unstated ethical approval |
| Question 10: Recommendations | *Yes*- The following must be discussed: Contributions to existing knowledge, identifies areas for future research, makes recommendations based on results |
|  | *Unsure*- only 2 items discussed |
|  | *No*- only 1 item discussed |
| Scoring system: |  |
| Yes: 1 point | High-quality paper: Scores 9–10 |
| Unsure: 0.5 points | Moderate-quality paper: Scores 7.5-9 |
| No: 0 points | Low-quality paper: Less than 7.5 |
|  | Exclude: Less than 6 |

**References**

BUTLER, A., HALL, H. & COPNELL, B. 2016. A guide to writing a qualitative systematic review protocol to enhance evidence‐based practice in nursing and health care. *Worldviews on Evidence‐Based Nursing,* 13**,** 241-249.

VAN GROOTEL, J., COLLET, R., VAN DONGEN, J., VAN DER LEEDEN, M., GELEIJN, E., OSTELO, R., VAN DER SCHAAF, M., WIERTSEMA, S. & MAJOR, M. 2024. Experiences with hospital-to-home transitions: perspectives from patients, family members and healthcare professionals. A systematic review and meta-synthesis of qualitative studies. *Disability and Rehabilitation***,** 1-14.
